# Metabolomic analysis of low and high biofilm-forming *Helicobacter pylori* strains

**DOI:** 10.1038/s41598-018-19697-0

**Published:** 2018-01-23

**Authors:** Eric Hong Jian Wong, Chow Goon Ng, Khean Lee Goh, Jamuna Vadivelu, Bow Ho, Mun Fai Loke

**Affiliations:** 1 Helicobacter Research Laboratory, Department of Medical Microbiology, Faculty of Medicine, University of Malaya, Kuala Lumpur, Malaysia; 2 Department of Microbiology and Immunology, Yong Loo Lin School of Medicine, National University of Singapore, Singapore, Singapore; 3 Departmemt of Medicine, Faculty of Medicine, University of Malaya, Kuala Lumpur, Malaysia; 4 Present Address: Singapore Precision Medicine Centre PTE LTD, Singapore, Singapore

## Abstract

The biofilm-forming-capability of *Helicobacter pylori* has been suggested to be among factors influencing treatment outcome. However, *H. pylori* exhibit strain-to-strain differences in biofilm-forming-capability. Metabolomics enables the inference of spatial and temporal changes of metabolic activities during biofilm formation. Our study seeks to examine the differences in metabolome of low and high biofilm-formers using the metabolomic approach. Eight *H. pylori* clinical strains with different biofilm-forming-capability were chosen for metabolomic analysis. Bacterial metabolites were extracted using Bligh and Dyer method and analyzed by Liquid Chromatography/Quadrupole Time-of-Flight mass spectrometry. The data was processed and analyzed using the MassHunter Qualitative Analysis and the Mass Profiler Professional programs. Based on global metabolomic profiles, low and high biofilm-formers presented as two distinctly different groups. Interestingly, low-biofilm-formers produced more metabolites than high-biofilm-formers. Further analysis was performed to identify metabolites that differed significantly (p-value < 0.005) between low and high biofilm-formers. These metabolites include major categories of lipids and metabolites involve in prostaglandin and folate metabolism. Our findings suggest that biofilm formation in *H*. *pylori* is complex and probably driven by the bacterium’ endogenous metabolism. Understanding the underlying metabolic differences between low and high biofilm-formers may enhance our current understanding of pathogenesis, extragastric survival and transmission of *H. pylori* infections.

## Introduction

Bacterial biofilm constitutes of a community of single or multiple species bacteria adhering onto any surface and encasing in an exopolysaccharide matrix^1–3^. The ability of unicellular bacteria to function together as a multicellular population through the formation of biofilm provides survival advantage to the bacterial population as a whole^2,4^ has its merit.

*Helicobacter pylori* is a bacterial pathogen that has been strongly associated with various gastric diseases ranging from chronic gastritis to mucosal-associated lymphoid tissue (MALT) lymphoma and gastric adenocarcinoma^5^. Interestingly, the organism has been demonstrated to form biofilm *in vitro*^6–11^. In addition, *H. pylori* biofilm has been demonstrated in the stomach of C57Bl/6J mice^6^ as well as the human stomach^7,10^. Furthermore, we recently demonstrated that *H. pylori* has the ability to form biofilm on vegetables, which is a common food source for human, potentially plays an important role in its survival and transmission of in the extragastric environment^12^. As biofilm has been demonstrated to decrease susceptibility of the biofilm-forming bacteria to antibiotics^13,14^, controlling and preventing biofilm formation by *H*. *pylori* may contribute to successful eradication of *H. pylori*. Recent studies unveiled the complexity the molecular mechanisms underlying *H*. *pylori* biofilm formation^15,16^. *H*. *pylori* biofilm formation has been found to involve the initiation of multiple mechanisms in flagella movement, bacterial virulence, signal transduction and regulation^16^, and regulated by the combined effect of multiple genetic factors^15^.

Study by Beale *et al*.^17^ on the biofilm metabolomics in water distribution revealed that through the analysis of the metabolites consumed or excreted as a result of metabolic activities, the biofilm activity could be elucidated. Metabolites are the results of the interaction of the microbial genome with its environment where the metabolites are an integral part of any cellular regulatory systems^18^. In addition, the study of metabolites enables the understanding of biofilm developmental process and bacterial molecular components which are important for establishing the mutual association of biofilm formation^19–22^.

Metabolomics has been widely applied to interrogate the free-floating planktonic and adhering biofilm lifestyles of various bacteria such as *Acinetobacter baumannii*^23^, *Salmonella* Typhimurium^24^, *Vibrio fischeri*^20^ and *Staphylococcus aureus*^22^. Importantly, Booth *et al*.^25^ using the metabolomic approach demonstrated that metabolic changes in response to copper exposure were found to be significantly different in *Pseudomonas fluorescens* biofilms compared to planktonic cultures. However, metabolomic differences between low and high biofilm-forming *H. pylori* have not been investigated. To better understand the factors involved in the biofilm formation of *H. pylori*, this study used the metabolomic approach via liquid chromatography/mass spectrometry (LC/MS). The data was then analyzed using MassHunter Qualitative Analysis software to identify the differences between metabolomics profiles of low and high biofilm-forming *H. pylori* clinical strains. We hypothesized that low and high biofilm-forming capability of *H. pylori* under a particular condition is determined by their endogenous metabolic capability. In general, biofilm formation could be readily detected by the third day in *in-vitro* cultures of high biofilm-forming *H. pylori* strains. Hence, in this study, bacterial metabolites of pre-biofilm *H. pylori* cultures (i.e. day 0 to 3) were analyzed to identify metabolites that may influence the initiation of biofilm formation.

## Results

### *H. pylori* biofilm

From our earlier studies, *H. pylori* clinical strain UM032, UM065, UM066, and UM067 were shown to be high biofilm-forming strains while UM038, UM054, UM084, and UM114 were low biofilm-forming strains^12,26^. The bacterial strains were cultured on chocolate blood agar plates, then the 3-day old *H. pylori* cultures were suspended in broth media for the development of biofilm (i.e. day 0). Biofilm formation was observed at the air-liquid interface of cultures of the high biofilm-forming stains but not in the low biofilm-forming stains by day 3. Scanning electron micrographs of the day 3 biofilm of a high biofilm-forming *H*. *pylori* strain are shown in Supplementary Fig. S1. Based on these findings, the metabolomic study was designed to study the differences in bacterial metabolism between high biofilm-forming strains and low biofilm-forming strains from day 0 to 3.

### Global metabolomics profile

**Figure 1:**
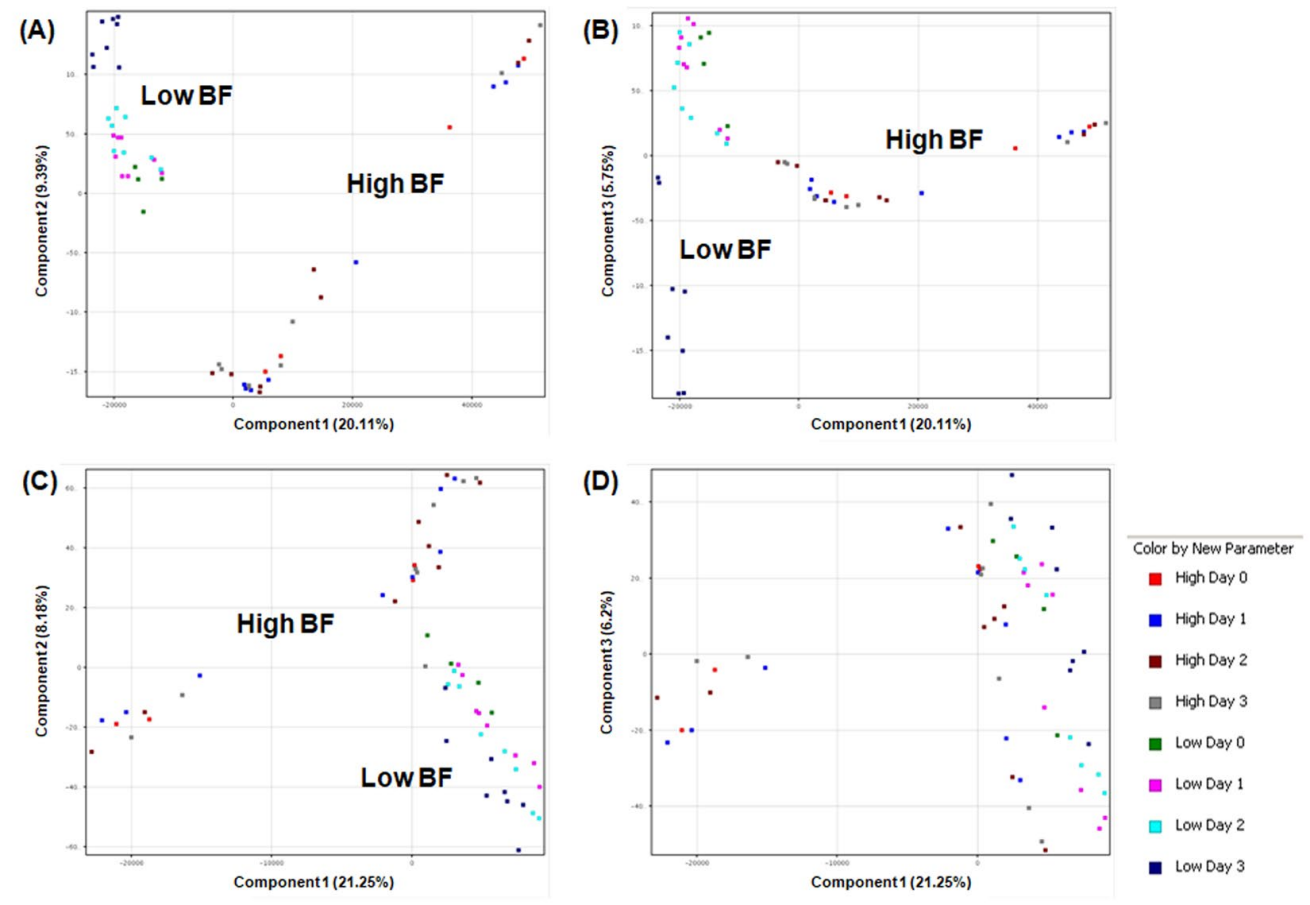
Two-dimensional principal component analysis (PCA) of 56 samples based on global metabolomic profiles. The PCAs were generated using MassHunter Mass Profiler Professional (MPP) software. There were 28 low and 28 high biofilm-forming samples. Independent replicates are available for Day 1 to 3 samples. Axes represent the principle components of the PCA and the numbers indicate the percentage of variance that is captured by each component. *H. pylori* strains formed distinct clusters on the basis of features acquired in positive ionization mode using (**A**) components 1 (20.11%) and 2 (9.39%), and components 1 (20.11%) and 3 (5.75%) (**B**). *H*. *pylori* strains formed distinct clusters on the basis of molecular features acquired in negative ion mode using components 1 (21.25%) and 2 (8.18%) (**C**) but not when components 1 (21.25%) and 3 (6.2%) was used (**D**). The scores shown by the axes scales are used to check data quality.

The unsupervised principal component analysis (PCA), which “condenses information contained in a large number of original variables into a smaller set of new composite dimensions with minimum loss of information”, was for quality control of samples^27^. When PCA using Euclidean distance was applied to compare the global metabolomic profiles (i.e. all molecular features without filtering) of the 8 individual strains, low and high biofilm-forming *H*. *pylori* strains partition into 2 well-separated groups based on their distinctive profiles on the PCA plots (Fig. 1A–C). The grouping of these *H. pylori* strains corresponds to their biofilm-forming capability, irrespective of age of culture. Replicates within a group should cluster together. On the other hand, the PCA plot generated based on compounds acquired in the negative ionization mode using principal components 1 and 3 failed to separate between low and high biofilm formers (Fig. 1D). Hence, clustering pattern shows that separation between low and high biofilm-formers was more differentiated based on positively-acquired compounds (Fig. 1A and B) compared to compounds acquired in negative ionization mode (Fig. 1C).

Using one-way ANOVA, a total of 1650 molecular features (1454 features were identified in the positive ionization mode and 196 features in the negative ionization mode) were identified to be significantly different (p-value < 0.005) between low and high biofilm-formers from day 0 to 3. After removing redundant molecular features that appeared in both positive and negative lists, 1468 features remained. Notably, 1434 features were increased in low biofilm-formers. In contrast, only 34 features were increased in high biofilm-formers. Thus, the metabolomes of low biofilm-forming *H. pylori* were demonstrated to be more diversity compared to high biofilm-forming *H. pylori*. Among these features, 351 features (330 features were increased in low biofilm-formers and only 21 features were increased in high biofilm-formers) matched metabolites in the Agilent METLIN Accurate Mass-Personal Metabolite Database and Library (AM-PCDL) database based on accurate mass, isotope ratios, abundances and spacing The 351 metabolites identified are listed in Supplementary Table S1. The mean number of metabolites per sample was 94 (30–176) for the high biofilm forming group and 317 (281–341) for the low biofilm forming group. The number of metabolites per low biofilm forming sample was significantly higher than the number of metabolites per high biofilm forming sample (p-value < 0.005).

**Figure 2:**
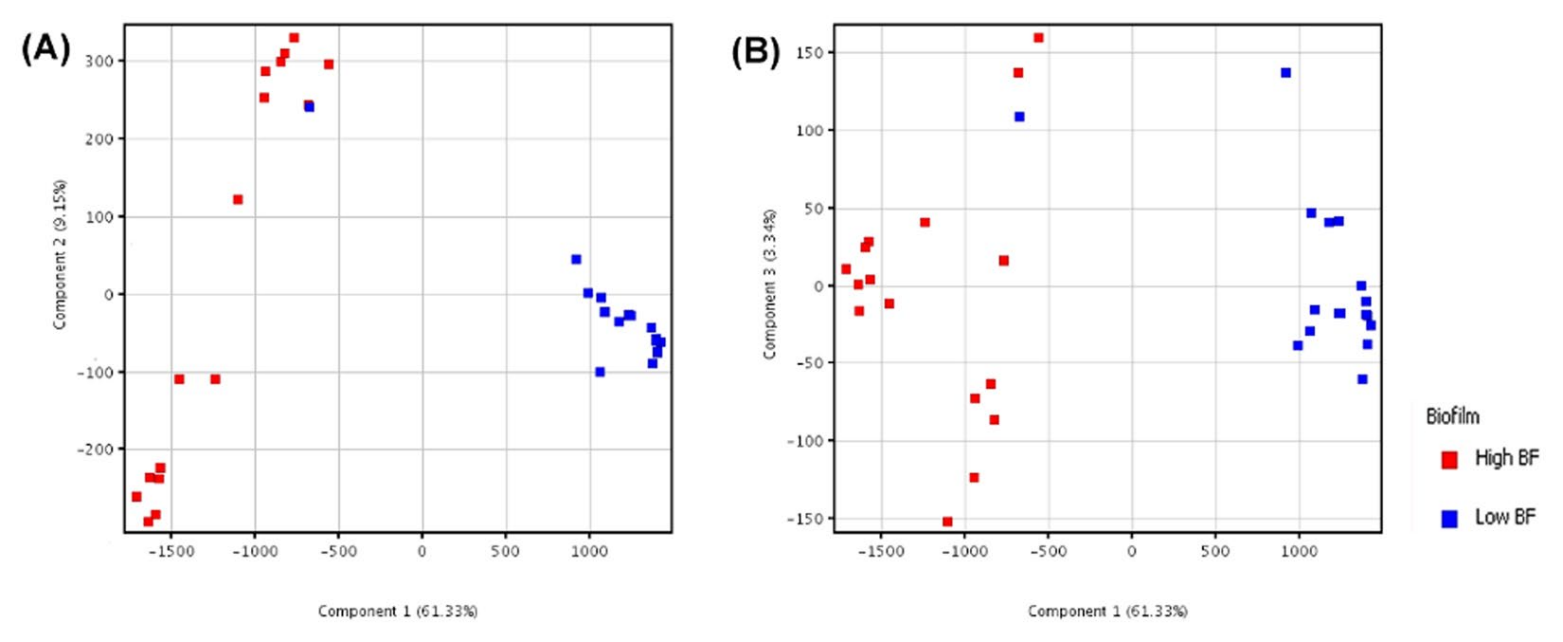
Two-dimensional principal component analysis (PCA) based on 351 significantly different metabolites. The PCAs were generated using MPP. Legend: red (high biofilm-formers, *N* = 28) and blue (low biofilm-formers, *N* = 28). Independent replicates are available for Day 1 to 3 samples. Axes represent the first three principle components of the PCA and the numbers indicate the percentage of variance that is captured by each component. (**A**) Shows the *H. pylori* strains separated based on components 1 (61.33%) and 2 (9.15%), while (**B**) shows the strains separated based components 1 (61.33%) and 3 (3.34%).

**Figure 3:**
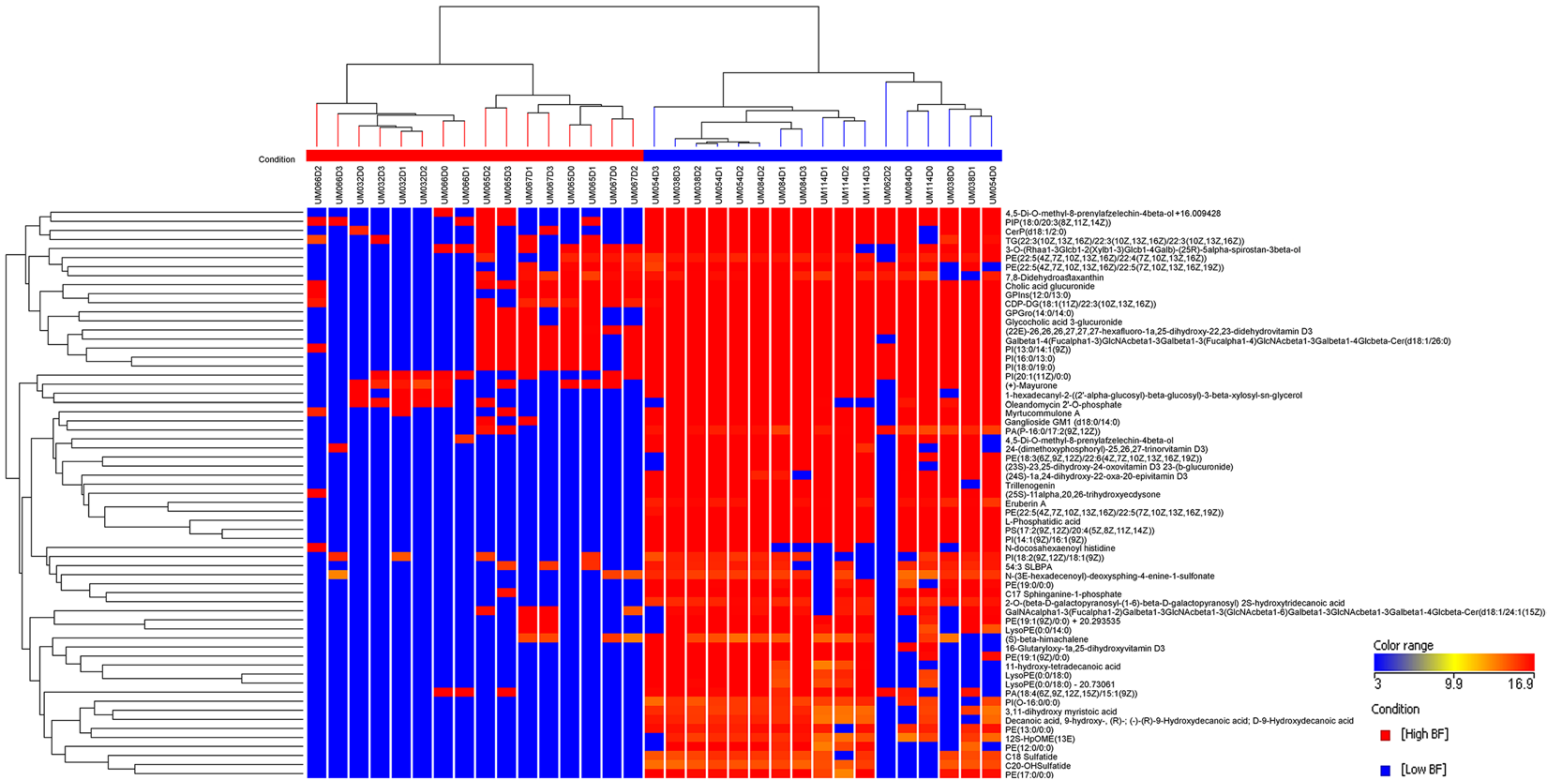
Heat map and clustering presenting metabolomic profiles of low and high biofilm-formers based on 65 significantly different lipids. The heat maps and clustering based on Euclidean distance were generated using MPP. Features with p-value < 0.005 were considered to be significantly different between low and high biofilm-formers. Details of these lipids are listed in Suppl. Table S1.

After statistically filtering out non-differential metabolites based on one-way ANNOVAR, metabolites acquired using positive and negative ionization modes were combined for further analysis. PCA plots of differential molecular features show that the metabolomic profiles of high biofilm-formers display a more spread out pattern suggesting that they were more heterogeneous in nature (Fig. 2). In contrast, low biofilm-formers, which clustered closer together on the PCA plots, were more homogenous. Similarly, the low and high biofilm-formers are clearly illustrated as two distinct groups in the heat map by the metabolites that were significantly different (p-value < 0.005) (Fig. 3).

Among the 351 metabolites identified, 65 significantly differentiating metabolites were lipids belonging to different categories. The heat map profile (Fig. 3) of these 65 lipids revealed that lipids were enriched in low biofilm-formers as compared to the high biofilm-formers. In contrast, few metabolites were increased in high biofilm-formers relative to low biofilm-formers. This finding indicates distinct differences between the metabolome of low and high biofilm-formers, which is consistent with the PCA plots. The metabolome of low biofilm-formers was also more complex than that of high biofilm-formers.

### Biofilm-associated metabolites and pathway analysis

Among metabolites that were enriched in low biofilm-formers were 65 lipids of different categories, comprising of 26 glycerophospholipids, 10 sterol lipids, 7 sphingolipids, 6 fatty acyls, 5 polyketides, 3 prenol lipids, 3 lysophosphatidylethanolamine, 2 phosphatidylglycerides, 2 glycerolipids, and a glycosphingolipid (Supplementary Table S2). Notably, (24S)-1α,24-dihydroxy-22-oxa-20-epivitamin D3, 16-glutaryloxy-1α,25-dihydroxyvitamin D3, 24-(dimethoxyphosphoryl)-25,26,27-trinorvitamin D3, (22E)-26,26,26,27,27,27-hexafluoro-1α,25-dihydroxy-22,23-didehydrovitamin D3, and (23S)-23,25-dihydroxy-24-oxovitamin D3 23-(β-glucuronide) are derivatives of vitamin D3.

In addition to lipids, non-lipid metabolites were also higher in low biofilm-formers. These include 5-oxoavermectin “2b” aglycone (KEGG C11954), avermectin B2b monosaccharide (KEGG C11957) and avermectin A2b monosaccharide (KEGG C11958), which are involved in the biosynthesis of 12-, 14- and 16-membered macrolides (map00522). Alkaloids, terpenoids, and triterpene glycosides were increased in low biofilm-formers.

Interestingly, N(6)-(octanoyl)lysine, a modified lysine amino acid that is normally found in acyl carrier protein, was also significantly higher in low biofilm formers (p-value = 6.49E-33). L-arginine (KEGG C00062), D-arginine (KEGG C00792) and p-coumaroylputrescine (a metabolite of arginine metabolism; KEGG C18326) were mapped to arginine and proline metabolism (map00330) and/or D-arginine and D-ornithine metabolism (map00522). Phenylacetaldehyde (KEGG C00601) and Phenylethylamine (KEGG C05332) were mapped to phenyalanine metabolism (map00360). Similarly, lignans (polyphenolic substances derived from phenylalanine) were also higher in low biofilm-formers.

Notably, L-Arginine (KEGG C00062), Cilastatin (KEGG C01675) and Neomycin B (KEGG C01737) were mapped to antibiotics biosynthesis (map00331, map 00524 and map01130). A sterol lipid, cholic acid glucuronide (KEGG C03033) and Indinavir (KEGG C07051) were mapped to bile secretion (map04976). These metabolites were also found in the low biofilm-formers but not the high-biofilm formers.

In contrast, 5-methyltetrahydrofolic acid (5-methyl-THF; KEGG: C00440; HMP: HMDB1396) was significantly increased in high biofilm-formers by 3.3-fold (p-value = 4.29E-07).

### *H. pylori* J99Δ*luxS* and J99Δ*cagY*

**Figure 4:**
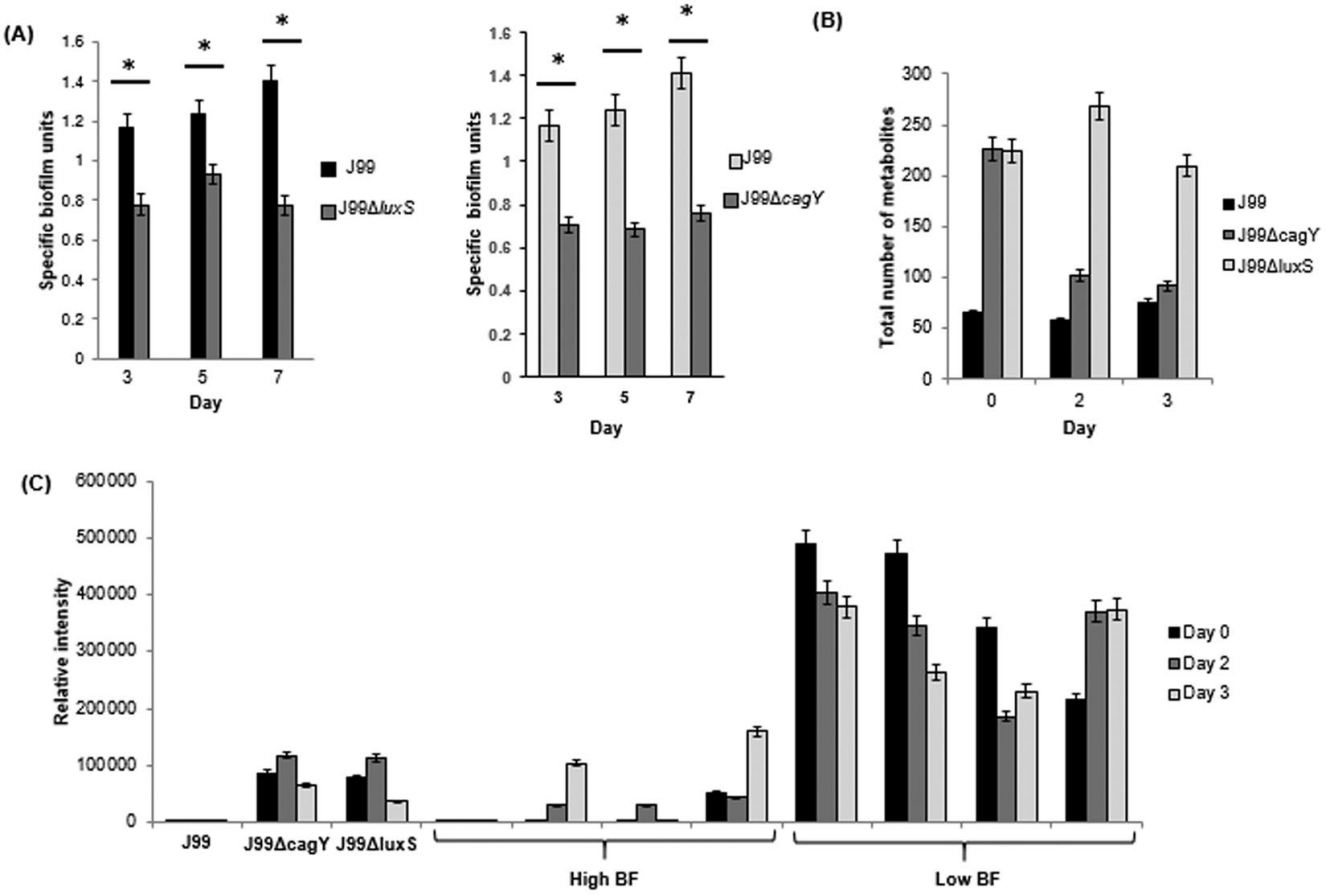
(**A**) Specific biofilm unit of wild-type *H. pylori* J99, J99Δ*luxS* and J99Δ*cagY* determined by crystal violet assay. (**B**) Comparison of number of metabolites detected by LC-MS per sample for *H. pylori* J99, J99Δ*luxS* and J99Δ*cagY*. *Represents p-value < 0.05 comparing between wild-type and mutants. (**C**) Comparing the relative abundance of PI-Cer(t18:0/18:0(2OH)) in *H. pylori* J99, J99Δ*luxS*, J99Δ*cagY*, high biofilm-forming clinical strains and low biofilm-forming strains.

*H. pylori* J99Δ*luxS* mutant strain was created in the laboratory of Associate Professor Hardie Kim at the University of Nottingham while J99Δ*cagY* mutant strain was generated as part of another study. Crystal violet assay was performed as described previously^12,26^. Both J99Δ*luxS* and J99Δ*cagY* mutant strains were found to have significantly reduced biofilm-forming abilities compared to wild-type J99 (p-value < 0.05; indicated by*) (Fig. 4A). Associated with reduced biofilm-forming abilities of the mutant strains was the observation of increased number of metabolites produced by the mutant strains compared to the isogenic wild-type J99 (p-value < 0.05; indicated by*) (Fig. 4B). Among the metabolites detected in only the mutant strains but not found in wild-type J99 was ceramide phosphoinositols (PI-Cer(t18:0/18:0(2OH))) or N-(2-hydroxyoctadecanoyl)-4R-hydroxysphinganine-1-phospho-(1′-myo-inositol) (LMP ID LMSP03030037), which is a phosphosphingolipid. Interestingly, PI-Cer(t18:0/18:0(2OH)) was high in low biofilm-forming strains but low or not detected in high biofilm-forming strains (Fig. 4C).

## Discussion

Metabolomics has been employed to investigate the differences between planktonic cultures and biofilms of various microbes^23,25^. This study has taken a step further to utilize metabolomics in distinguishing the metabolomes of *H*. *pylori* with low and high biofilm-forming capabilities. From day 0 to day 3, both high and low biofilm formers were still predominantly in the planktonic form. In contrast, for cultures after day 3, the metabolomic profile of high biofilm forming *H. pylori* is expected to be the combined effects of both planktonic and biofilm bacteria while the profile of low biofilm formers is due solely from planktonic bacteria. Therefore, only cultures from day 0 to day 3 were analyzed in order to minimize the confounding effect of biofilm bacteria in high biofilm formers. Comparative metabolomics of pre-biofilm forming cultures from day 0 to day 3 enable the identification of metabolites potentially associated with biofilm initiation and development. Our study showed distinct separation of high from low biofilm-formers based on PCA plots and identification of differential metabolites associated with these two groups of *H*. *pylori*. It also demonstrated the potential of metabolomics for use in the study of biofilm development.

It is not unexpected that low biofilm-formers probably have greater tendency to remain in their free-floating planktonic state than high biofilm-formers. The study shows that the low biofilm-forming *H*. *pylori* strains produced larger amount and more diverse types of metabolites indicating higher endogenous metabolic activities (especially lipid metabolism) compared to their high biofilm-forming counterparts regardless of age of cultures (day 0–3). Consistent with this observation, the inactivation of two genes (*luxS* and *cagY*) were demonstrated to reduce biofilm forming abilities and associated with increase in type of metabolites detected. Thus, metabolomics result in this study suggests that biofilm-forming capability of *H. pylori* strains may be influenced by the endogenous metabolic activities of the bacterium. It has been proposed earlier by Costerton *et al*.^3^ that adhered-bacteria (akin to biofilm-formers), are less metabolically active than planktonic bacteria.

The metabolites that were increased in the low biofilm-formers were of different categories of lipids, namely glycerophospholipids, sterol lipids, sphingolipids, fatty acyls, polyketides, prenol lipids, lysophosphatidylethanolamine, phosphatidylglycerides, glycerolipids, and glycosphingolipid. It is interesting to note that there has been no report of these lipids relating to *H. pylori* biofilm formation. However, an unpublished study carried out earlier in our lab (NUS) found that commercial full cream milk sustained the culturability of *H*. *pylori* for longer duration than low fat milk at 4 °C. *H. pylori* spiked into full cream milk remained culturable for as long as 72 hours while those in low fat milk completely loss their culturability after 4–8 hours at 4 °C (data not shown). Full cream milk contained 2- to 3-fold higher fat content compared to low fat milk. Consistently, BHI broth supplemented with fat extracted from milk was found to sustain the culturability of *H. pylori* for a longer period than just BHI alone at 4 °C. These findings indicate that lipids may contribute towards sustaining the viability of *H. pylori* or maintaining the bacterium in its planktonic state. Thus, endogenous biosynthesis of lipids by certain *H. pylori* strains may be associated with low biofilm-formation.

Phospholipids and sphingolipids are important components of cell membranes. These lipids (predominately long chain phospholipids and sphingolipids) are increased in low biofilm-formers indicating that the membrane of low biofilm-formers may have higher lipid stability and lower membrane fluidity. In addition, sphingolipids have previously been reported to inhibit the adherence of *Streptococcus mutans* to hydroxyapatite surfaces^28^. Hence, it is postulated that the presence of higher abundance of sphingolipids in low biofilm-formers may be unfavorable for bacteria-bacteria and bacteria-surface adherence necessary for the initiation of biofilm formation.

It has been established that many lipids (especially fatty acids) may be important in cell signaling that regulates a wide range of cellular functions^29^. These signals have been identified in both Gram-positive and Gram-negative bacteria as a unique chemical class of quorum sensing signals^29^. Various bacterial activities, such as in intra-species, inter-species and cross-kingdom communications that regulate bacterial growth, virulence, motility, polymer production, biofilm development, biofilm dispersion, and persistence, can be linked to fatty acid signaling^30–33^.

N(6)-(Octanoyl)lysine, which participates in the lipoic acid metabolism downstream of fatty acid biosynthesis, was found to be higher in low biofilm-formers. Degradation of acyl carrier protein or of lipoate-derivatized proteins will lead to free N(6)-(Octanoyl)lysine. lipoyl(octanoyl) transferase (EC 2.3.1.181). Interestingly, *Helicobacter pylori* has not been shown to posses lipoyl(octanoyl) transferase (EC 2.3.1.181) or lipoyl synthase (EC 2.8.1.8). However, it cannot be ruled that other enzyme(s) may perform similar functions to catalyze the transfer of endogenously produced octanoic acid from octanoyl-acyl-carrier-protein onto the lipoyl domains of lipoate-dependent enzymes.

Myrtucommulone A, a polyketide, was also found to be increased in low biofilm-formers compared to their high biofilm-forming counterparts. Myrtucommulone A is a lipophilic carboxylic acid with fatty acid-like character; they may bind to microsomal prostaglandin E synthase-1 (mPGES-1) as polyunsaturated fatty acids and inhibit the pro-tumorigenic prostaglandin E2 (PGE2) synthesis^34,35^. Prostaglandin is found to be synthesized during *Candida albicans* biofilm formation^36,37^. The inhibition of prostaglandin synthesis had greatly reduced fungal adhesion and detachment of mature biofilm^38,39^. Although *H. pylori* does not synthesize prostaglandin, it cannot be ruled out that myrtucommulone A synthesis by low biofilm-forming *H. pylori* strains may inhibit gastric prostaglandin synthesis, thereby influencing *H*. *pylori*-induced pathogenesis.

Results from the current study showed that biofilm formation in *H*. *pylori* may be influenced by its lipidome suggesting there may be a difference in membrane composition of low and high biofilm-formers. Notably, it has also been suggested that cholesterol may also play a vital role in virulence and contributes to the intrinsic antibiotic resistance of *H. pylori*^40^. Besides biofilm formation, the lipid composition of bacterial cells has also been shown to have an impact on antibiotic susceptibility^41^, probably due to changes in the penetrance of the membrane to antibiotics. Recently, Yonezawa *et al*.^13^ reported that *H. pylori* biofilm formation increases clarithromycin resistance and promotes associated-mutations more frequently than in planktonic cells. Therefore, *H. pylori* lipid biosynthesis pathways should be explored further to better understand the mechanism of antibiotic resistance and biofilm formation.

Higher abundance of multiple alkaloids, terpenoids, triterpene glycosides and polyphenolic compounds in low biofilm formers spurs our conjecture that these organic compounds may have a role in the initiation of biofilm formation. Alkaloids are synthesized from terpenoid and polyketide. This conjecture is supported by several reports demonstrating that plant and marine compounds with similar structure have anti-biofilm properties^42–45^. Thus, the potential use of phyto compounds to reduce *H. pylori* biofilm formation should not be overlooked.

5-Methyl THF, is a biologically active form of folic acid which was found to be increased in high biofilm-formers. In a nutritional complementation experiment by Lebeer *et al*.^46^, the introduction of folic acid helped to restore the biofilm-forming ability of *luxS* knockout mutant of *Lactobacillus rhamnosus* GG which has previously been shown to be defective in biofilm formation. Although eukaryotes are not known to possess the *luxS* gene, antifolates has also been shown to prevent *Cryptococcus* biofilm formation and inhibit mature biofilm^47^. These studies have highlighted the importance of folate metabolism in both prokaryotic and eukaryotic biofilm formation. Therefore, increase of 5-methyl THF in high biofilm-forming *H. pylori* strains may also demonstrate the importance of folate metabolism in *H. pylori* biofilm formation.

Currently, most of our understanding of bacterial biofilms comes from studying of biofilms cultured using established *in vitro* methods. However, there are expected to be significant differences between biofilms grown in the laboratory and *in vivo* biofilms found in infections. This is the major limitation of this study as the optimized laboratory condition for the culturing of *H. pylori* biofilm is not representative of the complex and dynamic condition of the human stomach or the environment, which is difficult to fully simulate. Thus, the metabolome of *in vitro H. pylori* biofilm may be different from that of biofilm found in the stomach or environment. Nevertheless, the findings of this study can form a rational basis for interrogating naturally occurring *H. pylori* and other bacterial biofilms in naturally occurring tissue and environmental samples.

## Conclusion

Biofilm formation in *H. pylori* at the initial growth stage is complex and is probably driven by its endogenous metabolism. The metabolism of low and high biofilm-forming *H. pylori* strains is different metabolically. Studying of the underlying metabolic processes that distinguish low and high biofilm-forming *H. pylori* will enhance our understanding of the possible role of biofilm formation in *H. pylori* pathogenesis, as well as extragastric survival and transmission of the organism. In addition, these metabolites may be potential anti-biofilm agents against the formation of *H. pylori* biofilm.

## Materials and Methods

### Culturing of *H. pylori*

*H. pylori* clinical strains used in this study were selected from the collection of the Helicobacter Research Laboratory at the University of Malaya (Kuala Lumpur, Malaysia). The biofilm-forming capability of each strain was determined through previous study^12,26^. Specific biofilm unit is the ratio of the absorbance values of crystal violet stained biofilm normalized against OD600 of the growth of corresponding planktonic bacteria. Specific biofilm unit was taken to be the amount of biofilm formed by the planktonic bacteria. The highest specific biofilm unit was determined for each of the strains. The bacterial strains were cultured on non-selective chocolate blood agar plates supplemented with 7% horse blood (Quad Five, MT, USA). The cultures were incubated at 37 °C for up to 3 days in a humidified 10% CO_2_ incubator (Thermo Scientific, MA, USA).

### Forming of *H. pylori* biofilm

The 3-day old *H. pylori* cultures were suspended in brain heart infusion (BHI) broth (Oxoid, Hampshire, UK) supplemented with 1% β-cyclodextrin (Sigma-Aldrich, MO, USA) and 0.4% yeast extract (Oxoid, Hampshire, UK). An aliquot of 10 ml of bacterial suspension containing 10^7^ CFU/ml of *H. pylori* was then inoculated into sterile DURAN® borosilicate glass bottle with screw cap (100 ml; Schott, Mainz, Germany) for the development of biofilm. BHI broth without *H. pylori* served as a negative control. The bottles were incubated in a humidified 10% CO_2_ incubator and monitored at day 0, 1, 2, and 3 for the presence of biofilm. The screw caps were left loose during incubation to allow for air circulation. Day 1, 2, and 3 cultures of each *H. pylori* strains were available in independent replicates.

### Extraction of metabolites

Biofilm culture was dislodged physically from the wall of the glass bottle and mixed with the planktonic culture before harvesting. As biofilm could readily be observed at the air-medium interface of high biofilm-forming strains cultures, this ensures that both representative low and high biofilm-forming samples were obtained. The bacterial cultures were harvested and washed 3 times at 10,000 × g for 10 minutes at room temperature with BHI broth. The bacterial suspensions were adjusted to OD_600 nm_ of 1.0 in BHI broth and 0.5 ml of bacterial suspensions were used for metabolite extraction. Bacterial pellets were processed according to the modified Bligh and Dyer extraction method^48^. In brief, 300 µl methanol-chloroform (2:1, v/v) was added to each bacterial pellet and vortexed for 1 minute followed by incubation for 1 hour at room temperature. Following which, 100 µl of chloroform and 100 µl of water were added to induce phase separation. The extract was left standing for 10 minutes at room temperature. The mixture was then centrifuged at 10,000 × g for 10 minutes at 4 °C. The upper aqueous and lower organic phases were collected and dried in Labconco Refrigerated Centrivap concentrator (Kansas City, MO, USA) at 4 °C. The dried samples were subsequently dissolved in 50 μl of acetonitrile/water (95:5, v/v) and pooled together for LC/MS analysis.

### LC/MS profiling of metabolites

Mass spectrometric analysis of the samples was performed on a 1260 Infinity Quaternary Liquid Chromatography system equipped with a 6540 Quadrupole Time-of-Flight mass spectrometer attached to a Dual Agilent Jet Stream Electrospray Ionization (Dual AJS ESI) ionization source (Agilent Technologies, CA, USA). Agilent MassHunter LC/MS Data Acquisition software (version B.05.01) was used for instrumental control and data collection. The samples were separated using the Agilent Zorbax Eclipse plus C18 Rapid Resolution High Throughput separation column (2.1 × 100 mm 1.8 μm) and analyzed in both positive and negative modes. In order to obtain a broad coverage of the metabolome, ionization was performed in both positive and negative modes. In the positive mode, the mobile phases used were water with 1% formic acid (A) and acetonitrile with 0.1% formic acid (B). In negative mode, the mobile phases were water with 1 mM ammonium fluoride and 0.1% formic acid (A) and acetonitrile (B). For both modes, a linear gradient was set to run from 2% to 98% B over 25 minutes, at 0.5 ml/min. In the event of the run, the injection volume was set at 3 μl with three injections were made for each sample. A typical sample ESI condition was set at voltage 3.0 kV, gas temperature 300 °C, drying gas 8 L/min, nebulizer 35 psig, VCap 3500 V, fragmentor 175 V and skimmer 65 V. The instrument was set to acquire over the m/z range of 100–1700 with an acquisition rate of 1 spectra/s. For positive ionization mode, two reference masses of (i) 121.0509 m/z and (ii) 922.0098 m/z were measured continuously while for negative ionization mode, the reference masses were (i) 112.9855 and (ii) 1033.9881.

### Data analysis and statistics

A two-pass feature extraction process was used to find molecular features from complex accurate mass data. Raw data was deconvoluted into individual chemical peaks by the MassHunter Qualitative Analysis software (Qual; version R.06.00) (Agilent Technologies, CA, USA) for Molecular Feature Extraction (MFE), a naïve or “untargeted” data-mining algorithm, which finds groups of co-variant ions for each unique feature in a chromatogram. Features with a minimum absolute abundance of 1000 counts within a defined mass accuracy (+/−5 ppm) were chosen. The data generated were subsequently imported into the MPP software (version B.12.61) for binning, aligning, and creating a consensus for each feature. The second pass used the list of consensus features and the “Find by Molecular Formula” algorithm in Qual for “targeted” feature re-extraction. This two-pass feature extraction approach improved the quality and accuracy of mass, retention time and abundance values for each candidate feature compared with the MFE only data mining. Finally, the data were imported into MPP again for annotation and statistical analysis. Using the baselining option of MPP, the abundance for each feature was normalized to the median abundance across all of the samples. Unsupervised principal component analysis (PCA) was performed with median centering and scaling to display the inherent variance between low and high biofilm-forming groups at different age of cultures. Statistical evaluation of the data was performed using one-way ANOVA. A cutoff value of p < 0.005 was considered statistically significant in one-way ANOVA, using the Benjamini and Hochberg False Discovery Rate (FDR) set to <1% for multiple testing corrections. Identification of the metabolites was done through the IDBrowser function of MPP by matching mined features based on accurate mass, isotope ratios, abundances and spacing against metabolites in the AM-PCDL database (version 5.0). Further statistical testing was carried out using the IBM SPSS Statistics (version 22) software.

## Electronic supplementary material

Article supplementary information

Supplementary Table S1
