# Supplementary material for: Metabolomic analysis of low and high biofilm-forming Helicobacter pylori strains
Source: Sci Rep. 2018 Jan 23;8:1409. doi: 10.1038/s41598-018-19697-0 (PMC5780479; doi:10.1038/s41598-018-19697-0)
Supplement: Supplementary file 1 — Article supplementary information [file 41598_2018_19697_MOESM1_ESM.pdf]

Metabolomic analysis of low and high biofilm-forming *Helicobacter pylori* strains

Eric Hong Jian Wong[1], Chow Goon Ng[2], Khean Lee Goh[3], Jamuna Vadivelu[1*], Bow Ho[2#a] and Mun Fai Loke[1,2*]

[1]Helicobacter Research Laboratory, Department of Medical Microbiology, Faculty of Medicine, University of Malaya, Kuala Lumpur, Malaysia.

[2]Department of Microbiology and Immunology, Yong Loo Lin School of Medicine, National University of Singapore, Singapore.

[3]Departmemt of Medicine, Faculty of Medicine, University of Malaya, Kuala Lumpur, Malaysia.

[*]These authors contributed equally to this work.

[#]Current address:

[a]Singapore Precision Medicine Centre PTE LTD, Singapore, Singapore.

Corresponding authors:

jamuna@ummc.edu.my (JV); lmunfai@gmail.com (MFL)

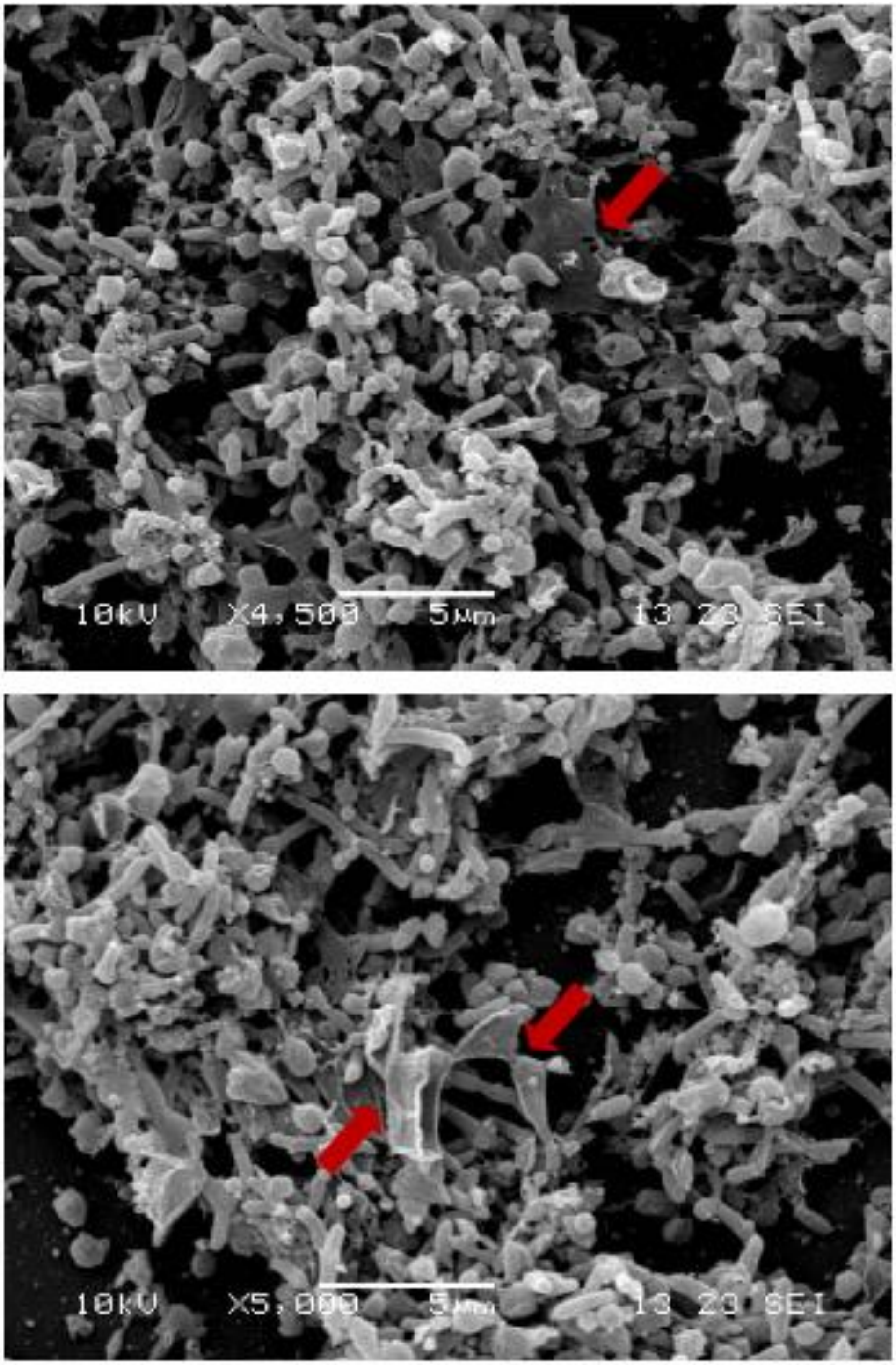

Supplementary materials Figure S1.

Representative scanning electron micrographs of 3 days old biofilm of a high biofilm former. *H. pylori* biofilm was grown on a glass cover slip at the air-medium interface. A mixture of spiral and coccoid forms was observed even as early as on day 3. Exopolysaccharide of biofilm can be seen as sheet-like structures (red arrow).

Suppl. Table S2.
Lipid metabolites increased in low biofilm-forming *H. pylori* strains.

Compound	p-value	Mean (HBF)	Mean (LBF)	FC	LMP ID	Category	Class
3,11-dihydroxy myristoic acid	1.49E-20	593	27910	47.07	LMFA01050081	Fatty Acyls [FA]	Fatty Acids and Conjugates [FA01]
Decanoic acid, 9-hydroxy-, (R)-; (-)-(R)-9-Hydroxydecanoic acid; D-9-Hydroxydecanoic acid	3.99E-15	713	31781	44.60	LMFA01050247	Fatty Acyls [FA]	Fatty Acids and Conjugates [FA01]
11-hydroxy-tetradecanoic acid	3.14E-13	1146	352320	307.55	LMFA01050174	Fatty Acyls [FA]	Fatty Acids and Conjugates [FA01]
2-O-(beta-D-galactopyranosyl-(1-6)-beta-D-galactopyranosyl) 2S-hydroxytridecanoic acid	7.36E-18	0	58832	NA	LMFA13010048	Fatty Acyls [FA]	Fatty acyl glycosides [FA13]
12S-HpOME(13E)	1.27E-17	500	32425	64.91	LMFA02000071	Fatty Acyls [FA]	Octadecanoids [FA02]
N-docosahexaenoyl histidine	2.57E-10	89598	1834555	20.48	LMFA08020134	Fatty Acyls [FA]	Fatty amides [FA08]
1-hexadecanyl-2-((2'-alpha-glucosyl)-beta-glucosyl)-3-beta-xylosyl-sn-glycerol + 9.871498	1.12E-11	72544	744020	10.26	LMGL04010001	Glycerolipids [GL]	Glycosylmonoradylglycerols [GL04]
TG(22:3(10Z,13Z,16Z)/22:3(10Z,13Z,16Z)/22:3(10Z,13Z,16Z))	5.11E-07	59569	238254	4.00	LMGL03012510	Glycerolipids [GL]	Triradylglycerols [GL03]
PS(17:2(9Z,12Z)/20:4(5Z,8Z,11Z,14Z))	0	0	993716	NA	LMGP03010300	Glycerophospholipids [GP]	Glycerophosphoethanolamines [GP02]
PE(22:6(4Z,7Z,10Z,13Z,16Z,19Z)/22:6(4Z,7Z,10Z,13Z,16Z,19Z))	7.22E-33	0	23280	NA	LMGP02010093	Glycerophospholipids [GP]	Glycerophosphoethanolamines [GP02]
PE(17:0/0:0)	2.17E-24	0	168497	NA	LMGP02050030	Glycerophospholipids [GP]	Glycerophosphoethanolamines [GP02]
PE(13:0/0:0)	7.06E-21	0	71731	NA	LMGP02050007	Glycerophospholipids [GP]	Glycerophosphoethanolamines [GP02]
PE(19:0/0:0)	2.00E-17	0	299715	NA	LMGP02050028	Glycerophospholipids [GP]	Glycerophosphoethanolamines [GP02]
PE(19:1(9Z)/0:0)	5.48E-12	60628	6771117	111.68	LMGP02050019	Glycerophospholipids [GP]	Glycerophosphoethanolamines [GP02]
PE(12:0/0:0)	2.26E-11	7055	318755	45.18	LMGP02050005	Glycerophospholipids [GP]	Glycerophosphoethanolamines [GP02]
PE(19:1(9Z)/0:0) + 20.293535	3.04E-10	441943	16126673	36.49	LMGP02050019	Glycerophospholipids [GP]	Glycerophosphoethanolamines [GP02]
L-Phosphatidic acid	1.56E-32	68571	1513793	22.08	-	Glycerophospholipids [GP]	-
PE(18:3(6Z,9Z,12Z)/22:6(4Z,7Z,10Z,13Z,16Z,19Z)) KEGG: C00350	2.95E-25	0	272400	NA	-	Glycerophospholipids [GP]	-
PI(14:1(9Z)/16:1(9Z))	2.83E-25	0	726571	NA	LMGP06010083	Glycerophospholipids [GP]	Glycerophosphoinositols [GP06]
PI(O-16:0/0:0)	7.77E-18	0	37055	NA	LMGP06060003	Glycerophospholipids [GP]	Glycerophosphoinositols [GP06]
PI(12:0/12:0)	1.71E-14	3293	31002	9.42	LMGP06010962	Glycerophospholipids [GP]	Glycerophosphoinositols [GP06]
GPIns(12:0/13:0)	1.30E-08	585814	2061801	3.52	LMGP06010003	Glycerophospholipids [GP]	Glycerophosphoinositols [GP06]

Compound	p-value	Mean (HBF)	Mean (LBF)	FC	LMP ID	Category	Class
PE(22:5(4Z,7Z,10Z,13Z,16Z)/22:5(7Z,10Z,13Z,16Z,19Z)) KEGG: C00350	1.83E-08	25422	75834	2.98	-	Glycerophospholipids [GP]	-
PE(22:5(4Z,7Z,10Z,13Z,16Z)/22:4(7Z,10Z,13Z,16Z)) KEGG: C00350	2.74E-07	40441	209646	5.18	-	Glycerophospholipids [GP]	-
PI(18:0/19:0)	9.31E-07	689744	1421660	2.06	LMGP06010285	Glycerophospholipids [GP]	Glycerophosphoinositols [GP06]
PI(16:0/13:0)	4.02E-07	2774287	6888244	2.48	LMGP06010160	Glycerophospholipids [GP]	Glycerophosphoinositols [GP06]
CDP-DG(18:1(11Z)/22:3(10Z,13Z,16Z))	2.67E-06	139250	501221	3.60	-	Glycerophospholipids [GP]	-
PI(20:1(11Z)/0:0)	2.57E-06	1012742	4399133	4.34	LMGP06050019	Glycerophospholipids [GP]	Glycerophosphoinositols [GP06]
PI(13:0/14:1(9Z))	2.06E-05	506672	1087936	2.15	LMGP06010038	Glycerophospholipids [GP]	Glycerophosphoinositols [GP06]
PG(14:0/0:0)	2.21E-13	1950	1162536	596.19	LMGP04050012	Glycerophospholipids [GP]	Glycerophosphoglycerols [GP04]
GPGro(14:0/14:0)	2.02E-08	103685000	27848484	3.72	LMGP04010005	Glycerophospholipids [GP]	Glycerophosphoglycerols [GP04]
54:3 SLBPA	4.13E-07	10372	53881	5.19	LMGP04090002	Glycerophospholipids [GP]	Glycerophosphoglycerols [GP04]
PA(P-16:0/17:2(9Z,12Z))	2.54E-17	14966	108881	7.28	LMGP10030011	Glycerophospholipids [GP]	Glycerophosphates [GP10]
PA(18:4(6Z,9Z,12Z,15Z)/15:1(9Z))	1.62E-09	39380	117197	2.98	LMGP10010428	Glycerophospholipids [GP]	Glycerophosphates [GP10]
PI(18:2(9Z,12Z)/18:1(9Z)) KEGG: C00626	6.58E-09	8723	49087	5.63	-	Phosphatidylglycerides	-
PIP(18:0/20:3(8Z,11Z,14Z)) KEGG: C00626	2.52E-07	2382962	6862759	2.88	-	Phosphatidylglycerides	-
LysoPE(0:0/18:0)	3.75E-14	0	937012	NA	-	Lysophosphatidylethanolamine	-
LysoPE(0:0/18:0) - 20.73061	3.95E-14	0	770976	NA	-	Lysophosphatidylethanolamine	-
LysoPE(0:0/14:0)	3.36E-09	111831	3358233	30.03	-	Lysophosphatidylethanolamine	-
Eruberin A	0	0	95856	NA	LMPK12020167	Polyketides [PK]	Flavonoids [PK12]
4,5-Di-O-methyl-8-prenylafzelechin-4beta-ol + 16.009428	3.40E-27	1535	1028453	669.96	LMPK12020217	Polyketides [PK]	Flavonoids [PK12]
4,5-Di-O-methyl-8-prenylafzelechin-4beta-ol	3.62E-15	4170650	14086948	3.38	LMPK12020217	Polyketides [PK]	Flavonoids [PK12]
Myrtucommulone A	3.92E-14	60446	230844	3.82	LMPK13070001	Polyketides [PK]	Aromatic polyketides [PK13]
Oleandomycin 2'-O-phosphate KEGG: C03796	2.83E-05	47528	202516	4.26	-	Polyketides [PK]	-
(+)-Mayurone	1.73E-07	549632	1977529	3.60	LMPR0103860002	Prenol Lipids [PR]	Isoprenoids [PR01]
(S)-beta-himachalene	1.55E-06	8470	61145	7.22	LMPR0103480006	Prenol Lipids [PR]	Isoprenoids [PR01]
7,8-Didehydroastaxanthin	4.37E-04	32395	86073	2.66	LMPR01070051	Prenol Lipids [PR]	Isoprenoids [PR01]

C20-OHSulfatide	33.27E-25	0	22787	NA	LMSP06020005	Sphingolipids [SP]	Acidic glycosphingolipids [SP06]
C18 Sulfatide	7.49E-21	0	31701	NA	LMSP06020001	Sphingolipids [SP]	Acidic glycosphingolipids [SP06]

Compound	p-value	Mean (HBF)	Mean (LBF)	FC	LMP ID	Category	Class
C17 Sphinganine-1-phosphate	5.69E-21	6105	133243	21.83	LMSP01050008	Sphingolipids [SP]	Sphingoid bases [SP01]
CerP(d18:1/2:0)	1.50E-11	2020975	10411116	5.15	LMSP02050014	Sphingolipids [SP]	Ceramides [SP02]
N-(3E-hexadecenoyl)-deoxysphing-4-enine-1-sulfonate	4.71E-08	4598	31874	6.93	LMSP00000003	Sphingolipids [SP]	Other Sphingolipids [SP00]
GalNAcalpha1-3(Fucalpha1-2)Galbeta1-3GlcNAcbeta1-3(GlcNAcbeta1-6)Galbeta1-3GlcNAcbeta1-3Galbeta1-4Glcbeta-Cer(d18:1/24:1(15Z))	4.77E-06	24778	82298	3.32	LMSP0504BF07	Sphingolipids [SP]	Neutral glycosphingolipids [SP05]
Galbeta1-4(Fucalpha1-3)GlcNAcbeta1-3Galbeta1-3(Fucalpha1-4)GlcNAcbeta1-3Galbeta1-4Glcbeta-Cer(d18:1/26:0)	3.29E-06	108831	339724	3.12	LMSP0504BC06	Sphingolipids [SP]	Neutral glycosphingolipids [SP05]
Trillenogenin	6.66E-33	0	408589	NA	LMST01080047	Sterol Lipids [ST]	Sterols [ST01]
(25S)-11alpha,20,26-trihydroxyecdysone	2.28E-17	50191	443781	8.84	LMST01010158	Sterol Lipids [ST]	Sterols [ST01]
3-O-(Rhaa1-3Glcb1-2(Xylb1-3)Glcb1-4Galb)-(25R)-5alpha-spirostan-3beta-ol	2.62E-04	240761	705792	2.93	LMST01080074	Sterol Lipids [ST]	Sterols [ST01]
(24S)-1α,24-dihydroxy-22-oxa-20-epivitamin D3	7.43E-25	0	267100	NA	LMST03020059	Sterol Lipids [ST]	Secosteroids [ST03]
16-Glutaryloxy-1α,25-dihydroxyvitamin D3	4.87E-20	0	3201782	NA	LMST03020634	Sterol Lipids [ST]	Secosteroids [ST03]
24-(dimethoxyphosphoryl)-25,26,27-trinorvitamin D3	1.08E-14	3798	179072	47.15	LMST03020072	Sterol Lipids [ST]	Secosteroids [ST03]
(22E)-26,26,26,27,27,27-hexafluoro-1α,25-dihydroxy-22,23-didehydrovitamin D3	1.71E-06	109708	265586	2.42	LMST03020082	Sterol Lipids [ST]	Secosteroids [ST03]
(23S)-23,25-dihydroxy-24-oxovitamin D3 23-(β-glucuronide)	1.58E-14	6387	140974	22.07	LMST05010020	Sterol Lipids [ST]	Steroid conjugates [ST05]
Glycocholic acid 3-glucuronide	8.95E-07	162523	496587	3.06	LMST05010023	Sterol Lipids [ST]	Steroid conjugates [ST05]
Cholic acid glucuronide	2.43E-05	4789619	9957613	2.08	LMST05010044	Sterol Lipids [ST]	Steroid conjugates [ST05]
Ganglioside GM1 (d18:0/14:0)	2.94E-25	53854	902019	16.75	-	Glycosphingolipid	-

FC: fold change; HBF: high biofilm-formers; LBF: low biofilm-formers; NA: Not applicable
1: below detection limit; MPP sets the lowest intensity to 1 for the purpose of calculating p-value
KEGG: Kyoto Encyclopedia of Genes and Genomes; HMP: Human Microbiome Project; LMP: LIPID MAPS Lipidomics
